# Performance of plasma separation cards for HIV drug-resistance genotyping in a resource-limited setting: comparison with plasma samples

**DOI:** 10.1093/jac/dkag279

**Published:** 2026-08-19

**Authors:** Thang Hong Pham, Hanh Thi Hong Ngo, Tram Thi Phuong Pham, Nhung Thi Hong Le, Mai Thi Nguyen, Tuan Anh Nguyen, Huyen Thi Do, Duong Nhu Tran, Lan Trong Phan, Yoann Madec

**Affiliations:** National Institute of Hygiene and Epidemiology (NIHE), Hanoi 100000, Vietnam; National Institute of Hygiene and Epidemiology (NIHE), Hanoi 100000, Vietnam; National Institute of Hygiene and Epidemiology (NIHE), Hanoi 100000, Vietnam; National Institute of Hygiene and Epidemiology (NIHE), Hanoi 100000, Vietnam; Vietnam Administration of Disease Prevention (VADP), Hanoi, Vietnam; National Institute of Hygiene and Epidemiology (NIHE), Hanoi 100000, Vietnam; Institute of Biotechnology, Vietnam Academy of Science and Technology, Hanoi, Vietnam; National Institute of Hygiene and Epidemiology (NIHE), Hanoi 100000, Vietnam; National Institute of Hygiene and Epidemiology (NIHE), Hanoi 100000, Vietnam; Epidemiology of Emerging Diseases, Institut Pasteur, Université de Paris, Paris F-75015, France

## Abstract

**Background:**

HIV drug resistance (HIVDR) threatens the effectiveness of antiretroviral therapy (ART) programmes in low- and middle-income countries. The Cobas Plasma Separation Card (PSC) is a recently developed specimen collection tool with potential to expand access to HIVDR genotyping in remote settings. This study evaluates PSC performance for HIVDR genotyping compared with plasma.

**Methods:**

Paired plasma and PSC specimens were selected from 250 people living with HIV (PLHIV) on ART ≥6 months with viral load (VL) ≥1000 copies/mL. HIVDR genotyping followed CDC Atlanta in-house protocol; resistance interpretation used the Stanford HIVdb algorithm. Paired sequences were compared for subtype, nucleotide similarity, mutation profile and drug-resistance classification.

**Results:**

Twenty paired specimens met inclusion criteria. Amplification of the *HIV-1 Pol* gene succeeded in 19/20 (95%) PSC samples. Of 19 paired sequences, nucleotide similarity was 98.5% (IQR 98.0%–99.3%). Eleven plasma samples showed 37 major drug-resistance mutations (MDM); 35 (94.6%) concordant MDM were identified in PSC. Discordances in four pairs were limited to mixed versus single bases and did not alter drug-resistance classification. Only one pair showed a clinically significant discordance (K103N detected in plasma but not initially in PSC).

**Conclusions:**

PSC demonstrated good concordance with plasma for HIVDR genotyping and has potential as an alternative specimen type for clinical practice and surveillance in resource-limited settings.

## Introduction

Vietnam’s HIV treatment programme has achieved significant progress, with 98% viral suppression among people on ART by end 2023.^1,2^ However, the effectiveness of these programmes is threatened by the emergence of HIVDR, which has increased steadily with expanding ART use.^3,4^ The WHO estimates that 26% of PLHIV initiating ART worldwide carry a virus resistant to first-line regimens.^5^ More systematic HIVDR testing could help minimize the spread of resistance and guide treatment decisions.^6^

Plasma is the gold standard for HIVDR genotyping, but collection, cold-chain maintenance and transport to centralized laboratories remain challenging in remote, resource-limited areas. Dried blood spots (DBS) offer logistical advantages but are affected by proviral DNA, and dry plasma spots (DPS) have shown poor amplification performance.^7,8^ The cobas PSC (Roche Molecular Diagnostics) is a built-in filtration system that separates plasma from whole blood and incorporates RNA-stabilizing reagents. Previous studies demonstrate good PSC performance for HIV VL testing,^9–12^ but its use for HIVDR genotyping has not previously been evaluated.

## Methods

### Sample selection

Paired plasma and PSC specimens were selected from stored samples of 250 PLHIV enrolled in a cross-sectional evaluation of PSC for VL testing.^13^ Inclusion criteria were: (i) plasma and PSC VL both ≥1000 copies/mL; and (ii) ART duration >6 months; and (iii) ≥3 PSC spots and ≥500 µL plasma available. Samples were collected between October 2022 and March 2023 and stored at −80°C at the National Reference Laboratory of HIV, NIHE, Hanoi, Vietnam.

### PSC preparation and extraction

PSC were prepared from EDTA whole blood within 24 hours of collection following the manufacturer’s instructions, left at room temperature for 7 days (to simulate field transfer), then stored at −80°C. One PSC spot (140 µL whole blood) was incubated in 2 mL NucliSENS lysis buffer (bioMérieux) and extracted on the eMAG system (50 µL elution volume). Plasma RNA was extracted from 140 µL using QIAamp Viral RNA kit (Qiagen, 50 µL elution volume).

### Genotyping and analysis

Reverse transcriptase PCR and nested PCR of the HIV-1 PR-RT gene region followed the CDC Atlanta/WHO protocol.^7,8^ Sanger sequencing was performed with BigDye Terminator v.3.1 on an ABI 3500 Genetic Analyzer. Sequences were assembled with RECall and aligned to *HXB2*. HIVDR mutations and subtypes were identified using the Stanford HIVdb algorithm and cross-checked with the ANRS algorithm. The HIVDR results on plasma were considered the gold standard. Paired plasma–PSC sequences were compared for subtype, nucleotide similarity, mutation profile and drug-resistance classification.

Ethics approval was granted by the IRBs of Institut Pasteur (IRB00006966) and NIHE (IRB-29/2021). All participants provided written informed consent before study enrolment after receiving detailed information about the study objectives, procedures and their rights as participants.

## Results

### Sample characteristics

Twenty paired plasma–PSC samples met inclusion criteria (Table 1). Patients were a median age of 37.5 years; 70% were male; median ART duration was 44.4 months. Median VL was 4.40 log copies/mL in plasma and 4.77 log copies/mL in PSC.

**Table 1.** Patient and sample characteristics of the 20 paired samples selected for HIVDR genotyping

Characteristics (*n* = 20)	Statistics
Age (years), median (IQR)	37.5 (29–41)
Male gender, *n* (%)	14 (70.0)
Duration on ART (months), median (IQR)	44.4 (10.5–49.5)
Storage duration until genotyping (months), median (IQR)	
* Plasma*	15.3 (13.5–15.6)
* PSC*	14.2 (14.7–16.8)
Storage temperature	−80°C (range −83°C to −77°C)
VL (log copies/mL), median (IQR)	
* Plasma*	4.40 (3.76–5.34)
* PSC*	4.77 (4.03–5.59)

### Amplification rates

Amplification of the *HIV-1 Pol* gene was successful in 19/20 (95%) PSC samples, while all 20 plasma samples were successfully amplified. For the single PSC sample that failed, amplification could not be retested because of insufficient remaining material. Among PSC samples, 14/19 were successfully amplified on the first attempt, four required a second amplification using 20 µL of RNA input instead of the initial 10 µL, and one required RNA re-extraction from one spot followed by amplification with 20 µL of RNA input before successful amplification was achieved.

### Subtype, nucleotide similarity and mutation profiles

All 19 paired sequences in the viral RT and PR regions were CRF01_AE. The median nucleotide similarity between paired PSC and plasma was 98.5% (IQR 98.0%–99.3%); 15/19 pairs (78.9%) exceeded 98% concordance. In 7/19 pairs (36.8%), no HIVDR mutations were detected in either specimen type. In seven further pairs (36.8%), identical major mutation profiles were observed in both specimen types (Table 2). In the remaining five pairs, discordances were observed: in four pairs the same mutations were present in both specimen types but differed only in mixed versus single base calls without any impact on drug-resistance classification. Notably, in three of these four pairs, the mixed-base call was detected in plasma but absent in PSC, whereas in one pair the mixed-base call was detected in PSC but not in plasma. Only in pair no. 15 was a clinically relevant discordance identified (K103N detected in plasma but not in PSC), resulting in a difference in predicted NNRTI susceptibility. Resequencing pair 15 using an increased RNA input (20 µL) from the PSC yielded K103N, matching the plasma result.

**Table 2.** Comparison of nucleotide similarity, subtype and HIVDR mutation profiles between 19 paired PSC and plasma samples

Patient	Sample	Subtype	Nucleotide similarity (%)	PI major mutation	NRTI mutation	NNRTI mutation
1	PSC	CRF01_AE	99.1	–	–	V106I
	Plasma	CRF01_AE		–	–	V106I
2	PSC	CRF01_AE	99.4	–	–	–
	Plasma	CRF01_AE		–	–	–
3	PSC	CRF01_AE	97.3	–	–	–
	Plasma	CRF01_AE		–	–	–
4	PSC	CRF01_AE	99.0	–	K65R, S68G, V75M, M184V	A98G, K103N, Y181C, G190A
	Plasma	CRF01_AE		–	K65R, S68G, V75M, M184V	A98G, K103N, Y181C, G190A
5*	PSC	CRF01_AE	97.7	–	–	K103**N**, K238**T**
	Plasma	CRF01_AE		–	–	K103**KN**, K238**RT**
6	PSC	CRF01_AE	98.8	–	M184V	A98AG, K103N, P225H
	Plasma	CRF01_AE		–	M184V	A98AG, K103N, P225H
7	PSC	CRF01_AE	98.7	–	–	K103N
	Plasma	CRF01_AE		–	–	K103N
8	PSC	CRF01_AE	99.7	–	M184V	–
	Plasma	CRF01_AE		–	M184V	–
9	PSC	CRF01_AE	98.1	–	–	–
	Plasma	CRF01_AE		–	–	–
10	PSC	CRF01_AE	99.8	–	–	–
	Plasma	CRF01_AE		–	–	–
11	PSC	CRF01_AE	99.5	–	K70KQ, M184I	K103N, K238T
	Plasma	CRF01_AE		–	K70KQ, M184I	K103N, K238T
12*	PSC	CRF01_AE	97.9	–	K65**KR**, S68G, M184MI	K103N, Y188C, M230ML
	Plasma	CRF01_AE		–	K65**R**, S68G, M184MI	K103N, Y188C, M230ML
13	PSC	CRF01_AE	99.4	–	–	–
	Plasma	CRF01_AE		–	–	–
14	PSC	CRF01_AE	98.3	M46L	–	–
	Plasma	CRF01_AE		M46L	–	–
15*	PSC	CRF01_AE	98.0	–	V75M	**–**
	Plasma	CRF01_AE		–	V75M	**K103N**
16	PSC	CRF01_AE	95.9	–	–	–
	Plasma	CRF01_AE		–	–	–
17*	PSC	CRF01_AE	97.8	–	–	K103**N**
	Plasma	CRF01_AE		–	–	K103**KN**
18	PSC	CRF01_AE	99.1	–	–	–
	Plasma	CRF01_AE		–	–	–
19*	PSC	CRF01_AE	98.7	–	K65**R**, T69del, V75M	V106**M**, V179**D**, Y181**C**
	Plasma	CRF01_AE		–	K65**KR**, T69del, V75M	V106**VM**, V179**VD**, Y181**YC**

Mixed bases are indicated by double-letter notation (e.g. K103KN). ‘–’ denotes no mutation detected.

Asterisks (*) indicate pairs with mutation discordances, bold values indicate the mutations that differ between the pair samples.

Overall, 37 MDM were identified across all plasma samples and 35 (94.6%) concordant MDM were identified in PSC.

## Discussion

To our knowledge, this is the first evaluation of the cobas PSC for HIVDR genotyping. Our findings indicate that DPS obtained from the PSC provides near-identical HIVDR results to plasma, with a 95% amplification rate and 94.6% MDM concordance at the ≥1000 copies/mL threshold. These results compare favourably with DBS-based studies conducted in Asia and Africa, which reported amplification rates of 78%–100%^14,15^ and exceed the poor performance previously documented for conventional DPS.^16^ The improved performance of PSC over DPS probably reflects its built-in filtration mechanism, RNA-stabilizing reagents and calibrated volume (140 µL per spot).

The high nucleotide similarity (98.5%) between paired specimens is comparable to the 98.8% concordance reported by a US CDC laboratory for DBS.^14^

This study demonstrated a high concordance in HIVDR mutation profiles between plasma and PSC specimens, with 94.6% (35/37) of all mutations detected at first-pass sequencing. The two mutations present in plasma but absent in PSC—K238R (pair 5) and K103N (pair 15)—carried distinct clinical weights: K238R is a common polymorphism without impact on NNRTI susceptibility, while K103N represents the sole clinically relevant discordance, conferring markedly reduced susceptibility to Nevirapine and Efavirenz. Both discordances probably reflect differences in viral RNA template abundance between specimen types, as PSC prepared from 140 µL of whole blood yields a lower effective plasma volume than the 140 µL of plasma used directly for sequencing. Consistent with this, resequencing pair 15 with increased RNA input from the PSC resolved the discordance, suggesting that optimizing extraction volume is a straightforward strategy to improve PSC sensitivity. Of the four mixed-base call discordances, however, none affected resistance classification. These discordances followed a directional pattern as they were more frequently identified in plasma than PSC (3/4 pairs), again consistent with higher template abundance in plasma rather than an intrinsic limitation of PSC-based sequencing. Collectively, these findings support the clinical equivalence of PSC for HIVDR genotyping.

Unlike DBS, PSC contains only plasma-derived material, avoiding proviral DNA contamination from peripheral blood mononuclear cells, a known confounder of DBS-based HIVDR genotyping.^17,18^ This property makes PSC more representative of circulating viral quasispecies.

This study was limited by its small convenience sample size, inclusion of only CRF01_AE viruses and the use of PSC specimens prepared and stored under controlled conditions. Further studies are needed to evaluate performance at lower VLs, across diverse HIV-1 subtypes and under field storage and transportation conditions. Nevertheless, PSC showed promising performance for HIVDR genotyping in patients failing ART and may facilitate HIVDR testing in resource-limited settings.

### Conclusions

The cobas PSC showed good concordance with plasma for HIVDR genotyping among ART-treated individuals with virological failure (VL >1000 copies/mL). These findings support its potential as a feasible alternative specimen type for HIVDR testing and surveillance, particularly where plasma collection and transport are challenging. Further studies are needed to validate PSC performance under field conditions and at lower VLs.

## Data Availability

The datasets used and/or analysed during the current study are available from the corresponding author on reasonable request.
